# Olaparib Blocks Oxidative Stress‐Dependent NLRP3 Inflammasome in Pediatric Acute Lung Injury by Metabolic Reprogramming via AMPK‐Driven Autophagic Flux

**DOI:** 10.1002/kjm2.70275

**Published:** 2026-10-09

**Authors:** Fen Gong, Mei‐Ling Yu, Zheng‐Rong Chen, Mei Li, Xu Sang, Yong‐Dong Yan

**Affiliations:** ^1^ Department of Respiratory Medicine Children's Hospital of Soochow University Jiangsu Province China; ^2^ Department of Pediatrics The Second Affiliated Hospital of Bengbu Medical University Bengbu Anhui People's Republic of China; ^3^ Department of Pharmacy The First Affiliated Hospital of Bengbu Medical University Bengbu Anhui People's Republic of China; ^4^ Pediatrics Research Institute Children's Hospital of Soochow University Suzhou P. R. China; ^5^ Deputy Chief Physician of Pediatrics First Affiliated Hospital of Bengbu Medical University Bengbu Anhui People's Republic of China

**Keywords:** AMPK, metabolic reprogramming, NLRP3 inflammasome, olaparib, pediatric acute lung injury

## Abstract

Pediatric acute lung injury (ALI) is a life‐threatening syndrome defined by dysregulated pulmonary inflammation and impaired alveolar gas exchange, with limited therapeutic interventions. This study aims to evaluate the therapeutic efficacy and mechanistic basis of Olaparib, a clinically approved PARP inhibitor, using in vitro and in vivo models of pediatric ALI. In LPS/ATP‐stimulated THP‐1‐derived macrophages, Olaparib induced metabolic reprogramming, as indicated by a reduced NAD^+^/NADH ratio and an elevated AMP/ATP ratio, leading to activation of the AMPK/ULK1 signaling pathway. This activation enhanced autophagic flux, as evidenced by increased LC3‐II conversion, p62 degradation, and analysis of the mRFP‐GFP‐LC3 reporter. Enhanced autophagy was associated with attenuation of oxidative stress, including reductions in mitochondrial and intracellular reactive oxygen species and malondialdehyde levels, and suppression of NLRP3 inflammasome activation, as evidenced by decreased cleaved caspase‐1, IL‐1β, and IL‐18. In a juvenile murine model of LPS‐induced ALI, Olaparib administration reduced histopathological lung injury, inflammatory cell infiltration, and pulmonary edema. It also restored alveolar–capillary barrier integrity by upregulating junctional proteins, including ZO‐1, claudin‐5, and VE‐cadherin, and reduced apoptosis. The protective effects of Olaparib on autophagy, oxidative stress, inflammasome activity, and lung tissue repair were attenuated by pharmacological inhibition of AMPK using compound C. In conclusion, these findings demonstrate that Olaparib ameliorates pediatric ALI through AMPK‐dependent induction of autophagic flux via metabolic reprogramming, attenuating oxidative stress and inhibiting NLRP3 inflammasome activation. These results support the therapeutic potential of Olaparib in pediatric ALI.

## 1 Introduction

Acute lung injury (ALI) and its severe form, acute respiratory distress syndrome (ARDS), are critical conditions characterized by acute pulmonary inflammation, impaired gas exchange, and hypoxemia, frequently progressing to respiratory failure [1]. ALI/ARDS affects more than 3 million individuals worldwide each year, with pediatric patients presenting distinct challenges due to developmental immaturity of the lungs and dysregulated immune responses [2]. In children, reduced surfactant production, diminished functional residual capacity, and increased vulnerability to ventilator‐induced lung injury during mechanical ventilation, the current standard of care, contribute to disease severity [3]. Corticosteroids are commonly administered to control inflammation; however, their use in pediatric populations is limited by significant adverse effects, including growth suppression and increased susceptibility to infections [4, 5]. These complications are further intensified by the immaturity of endogenous antioxidant systems, which promotes a self‐amplifying cycle in which oxidative stress disrupts alveolar–capillary barrier integrity and activates pro‐inflammatory signaling pathways, aggravating lung injury [6]. Pediatric ARDS affects approximately 3% of patients in the pediatric intensive care unit (PICU) and is associated with a mortality rate of 17% [7], underscoring the urgent need for targeted therapeutic strategies that address the distinct pathological characteristics of pediatric ALI.

The NACHT, LRR, and PYD domains‐containing protein 3 (NLRP3) inflammasome is a multiprotein complex composed of the sensor molecule NLRP3, the adaptor apoptosis‐associated speck‐like protein containing a CARD (ASC), and the effector protease caspase‐1. As a central mediator of ALI pathophysiology, excessive activation of the NLRP3 inflammasome induces a hyperinflammatory state that aggravates tissue damage and contributes to severe complications in ARDS [8]. Although pharmacological inhibition of NLRP3, such as with MCC950, demonstrates efficacy in preclinical ALI models [9], clinical translation remains constrained by the risks of immunosuppression and off‐target effects [10]. Pediatric patients with immunocompromised status are particularly susceptible to both the onset and increased mortality associated with ARDS [11], highlighting the need for approaches that selectively modulate inflammasome activity rather than completely inhibit it.

Autophagy is an evolutionarily conserved intracellular degradation pathway that facilitates the clearance of damaged cellular components and supports their recycling for energy production and biosynthesis. This process functions as a fundamental innate immune mechanism against intracellular pathogens [12]. Augmentation of autophagy has been shown to restore immune homeostasis and attenuate respiratory dysfunction associated with ALI/ARDS [13]. Impairment of autophagy promotes sustained activation of the NLRP3 inflammasome, positioning autophagy as a critical regulator of inflammasome signaling [14]. Through the selective removal of NLRP3 activators, including damage‐associated molecular patterns (DAMPs), inflammasome components, and pro‐inflammatory cytokines, autophagy limits inflammatory responses. Inflammasome signaling, in turn, modulates autophagic processes, establishing a regulatory pathway that maintains the balance between host defense and inflammatory injury [15]. Autophagy‐mediated regulation of the NLRP3 inflammasome constitutes a promising therapeutic target for pediatric‐specific intervention strategies.

Olaparib, a clinically approved poly(ADP‐ribose) polymerase (PARP) inhibitor widely used in BRCA‐mutant cancers, has demonstrated protective effects in lipopolysaccharide (LPS)‐induced ALI models by attenuating inflammatory responses and oxidative stress [16, 17]. PARP‐1 acts as a DNA damage sensor activated under diverse cellular stress conditions, while its substrate NAD^+^ functions as an essential coenzyme in redox reactions and as a regulator of non‐redox enzymes, including sirtuins and CD38 [18]. Beyond its canonical role in DNA repair, Olaparib‐mediated PARP inhibition modulates cellular energy homeostasis [19]. Under inflammatory conditions, Olaparib alters NAD^+^ metabolism and the AMP/ATP ratio, leading to activation of AMP‐activated protein kinase (AMPK). This key metabolic sensor orchestrates adaptive responses to cellular stress [20]. Activation of AMPK induces autophagy via phosphorylation of unc‐51‐like autophagy activating kinase 1 (ULK1) and modulation of mammalian target of rapamycin (mTOR) signaling, resulting in the initiation of a cytoprotective metabolic–autophagic program [21]. This integrated mechanism supports the potential application of Olaparib as a therapeutic strategy for pediatric ALI by targeting metabolic imbalance and defective autophagic regulation associated with disease progression.

On the basis of the above fact, this study aims to evaluate the therapeutic potential of Olaparib in LPS‐induced 3–4‐week‐old C57BL/6J mice and LPS/ATP‐stimulated THP‐1‐derived macrophages, with emphasis on its effects on metabolic reprogramming, AMPK‐mediated autophagy, oxidative stress, NLRP3 inflammasome activation, and cell death. It is hypothesized that Olaparib ameliorates pediatric ALI. The study is designed to identify a therapeutic strategy for pediatric ALI by targeting the NLRP3–reactive oxygen species (ROS)–autophagy pathway through modulation of cellular metabolism.

## 2 Materials and Methods

### 2.1 Ethical Statement

All animal experiments were approved by the Ethical Committee of Children's Hospital of Soochow University (No. 2025CS110) and were performed in accordance with the National Institutes of Health Guide for the Care and Use of Laboratory Animals.

### 2.2 Animals

Male C57BL/6J mice (3–4 weeks old) were obtained from Jiangsu Qinglongshan Biotechnology Co. Ltd. (China) and maintained in Plexiglas cages (4 mice per cage) under controlled environmental conditions (12‐h light/dark cycle, 22°C ± 2°C, 60% ± 5% humidity). Animals had ad libitum access to food and water. After a 1‐week acclimatization period, mice were randomly allocated into five experimental groups (*n* = 6 per group): sham, LPS, LPS + Olaparib (50 mg/kg), LPS + Olaparib (100 mg/kg), and LPS + Olaparib (100 mg/kg) + compound C (20 mg/kg).

The sham group received intraperitoneal (*i.p*.) injections of normal saline. The LPS group was administered 0.5 mg/kg LPS (HY‐D1056, MedChemExpress, Shanghai, China) dissolved in 0.9% saline, followed by a single *i.p*. injection of normal saline 1 h later [22]. Treatment groups received 0.5 mg/kg LPS *i.p*., followed 1 h later by a single *i.p*. injection of Olaparib (99.98% purity; HY‐10162, MedChemExpress) at either 50 or 100 mg/kg, dissolved in a mixture of dimethyl sulfoxide (DMSO) and 10% 2‐hydroxypropyl‐β‐cyclodextrin in saline. In the combination group, compound C (20 mg/kg; 99.10% purity; HY‐13418A, MedChemExpress) was co‐administered with Olaparib (100 mg/kg) 1 h after LPS injection. At 24 h post‐LPS administration, mice were euthanized via cervical dislocation under pentobarbital sodium anesthesia (100 mg/kg, *i.p*.). The trachea was exposed and cannulated for bronchoalveolar lavage fluid (BALF) collection. Following lavage, the lungs were excised for further analysis. The left lung lobe was fixed in 10% neutral‐buffered formalin for histological assessment, while the right lung was dissected into lobes: the upper and middle lobes were snap‐frozen for protein and biochemical analyses, and the lower lobe was used for the determination of the wet‐to‐dry weight ratio.

### 2.3 Lung Wet/Dry Ratio

Lung tissues were harvested 24 h after LPS administration, and the wet weight was measured immediately. For dry weight determination, tissues were incubated at 60°C for 24 h until a constant weight was achieved. The wet‐to‐dry weight ratio was calculated as an index of pulmonary edema.

### 2.4 Hematoxylin–Eosin Staining

Left lung tissues were fixed in 10% neutral‐buffered formalin at room temperature for 48 h, then dehydrated through a graded ethanol series, cleared in xylene, and paraffin‐embedded. Serial sections (4 μm) were prepared using a microtome, mounted on glass slides, and dried overnight at 37°C. Sections were deparaffinized in xylene, rehydrated through descending ethanol gradients, and rinsed in distilled water before staining with a commercial hematoxylin and eosin kit (H&E; C0105M, Beyotime, Shanghai) according to the manufacturer's protocol. Stained sections were then dehydrated, cleared in xylene, and visualized using a DP71 light microscope (Olympus, Tokyo, Japan). Histopathological evaluation was performed using a validated semi‐quantitative scoring system by two blinded pathologists [23]. Lung injury was assessed across four parameters: inflammatory cell infiltration, alveolar hemorrhage, alveolar edema, and interstitial edema, each graded on a scale from 0 (normal) to 3 (severe). The total lung injury score was obtained by summing individual parameter scores.

### 2.5 Immunohistochemical Staining

Paraffin‐embedded tissue sections (4 μm) were mounted on poly‐l‐lysine‐coated glass slides, followed by deparaffinization and rehydration through a graded series of solvents. Antigen retrieval was performed by heat induction in 10 mM sodium citrate buffer (pH ~6.0) using a microwave at 95°C for 15 min. Sections were allowed to cool to room temperature and rinsed with phosphate‐buffered saline (PBS). Endogenous peroxidase activity was quenched by incubation with 3% H₂O₂ in methanol for 20 min at room temperature, followed by blocking with 5% normal goat serum in PBS for 1 h at room temperature. The sections were then incubated overnight at 4°C with primary antibodies against F4/80 (ab300421, 1:5000; Abcam) and 4‐hydroxy‐2‐nonenal (4‐HNE; ab48506, 1:25; Abcam). After three PBS washes (5 min each), the slides were incubated with horseradish peroxidase (HRP)‐conjugated secondary antibodies (1:500) for 1 h at room temperature. Immunoreactivity was visualized using 3,3′‐diaminobenzidine (DAB; P0203, Beyotime) for 5 min, followed by hematoxylin counterstaining for 2 min. Following dehydration and mounting, stained sections were examined under a light microscope. Five randomly selected fields per lung section were imaged, and staining intensity was quantified using ImageJ (v1.53) by determining the percentage of the field area stained in each field.

### 2.6 Terminal Deoxynucleotidyl Transferase dUTP Nick End Labeling (TUNEL)

Left lung tissues were fixed in 4% paraformaldehyde for 24 h at 4°C, followed by dehydration through a graded ethanol series, clearing in xylene, paraffin embedding, and sectioning into 4‐μm‐thick slices using a microtome. TUNEL staining was performed using a TUNEL apoptosis detection kit (40306ES50, Yeasen, Shanghai) according to the manufacturer's protocol. Following deparaffinization and rehydration, tissue sections were permeabilized with proteinase K for 15 min at 37°C and washed twice with PBS. The sections were then incubated with a TdT enzyme and fluorescein‐dUTP reaction mixture for 60 min at 37°C in a humidified dark chamber. After three PBS washes, nuclei were counterstained with DAPI (1 μg/mL; C1002, Beyotime) for 10 min at room temperature. Slides were then washed three times with PBS and mounted using an anti‐fade mounting medium. Fluorescent images were acquired using a laser scanning confocal microscope (FluoView FV1000, Olympus). Five randomly selected fields per lung section were imaged. Apoptotic activity was quantified by calculating the ratio of TUNEL‐positive area to total tissue area using ImageJ software (v1.53), and results were expressed as percentage TUNEL‐positive area.

### 2.7 Myeloperoxidase (MPO) Activity Assay

MPO levels were quantified using a commercial Mouse MPO ELISA kit (BES1563K, BIOSEN, Shanghai) according to the manufacturer's instructions. Lung tissues (30 mg) were homogenized in 1 mL of ice‐cold PBS supplemented with protease inhibitors, followed by centrifugation at 12,000*g* for 20 min at 4°C. The resulting supernatants were collected for analysis. BALF samples were centrifuged at 500*g* for 10 min to remove cellular debris before assay. Standards and samples were added to antibody‐coated wells and incubated for 2 h. This was followed by incubation with biotinylated detection antibody for 1 h. After washing, streptavidin–HRP was added, and color development was achieved using tetramethylbenzidine (TMB) substrate. The reaction was terminated by adding 2 M H_2_SO_4_, and absorbance was measured at 450 nm using a SpectraMax i3x microplate reader (Molecular Devices). MPO concentrations were calculated from the standard curve and normalized to total protein content determined by bicinchoninic acid (BCA) assay.

### 2.8 Cell Culture

The human monocytic cell line THP‐1 (CL‐0233, Procell, Wuhan, China) was maintained in RPMI‐1640 medium supplemented with 10% fetal bovine serum (PM150110B, Procell), 100 U/mL penicillin, and 0.1 mg/mL streptomycin (C0222, Beyotime) at 37°C in a humidified atmosphere containing 5% CO_2_. For macrophage differentiation, THP‐1 cells were seeded in 6‐well plates at a density of 1 × 10^6^ cells per well and incubated for 24 h, followed by stimulation with 50 ng/mL phorbol 12‐myristate 13‐acetate (PMA; HY‐18739, MedChemExpress) for 24 h. Cells were then allowed to rest for 48 h to promote differentiation. THP‐1‐derived macrophages were washed twice with fresh medium before further experimental procedures.

### 2.9 Cell Treatment

THP‐1‐derived macrophages were seeded in 6‐well plates at a density of 5 × 10^5^ cells per well and incubated for 24 h before treatment. Olaparib was prepared as a 10 mM stock solution in DMSO and diluted in complete culture medium to the indicated working concentrations (5, 10, or 20 μM), ensuring a final DMSO concentration of 0.1% (v/v). Cells were pretreated with Olaparib for 1 h before stimulation. Macrophages were primed with LPS (100 ng/mL) for 4 h, followed by activation with ATP (5 mM; HY‐B2176, MedChemExpress) for 1 h. For pathway inhibition experiments, cells were pretreated with compound C (10 μM) for 24 h before LPS stimulation to inhibit AMPK signaling. Autophagy was inhibited using 3‐methyladenine (3‐MA; 5 mM; HY‐19312, MedChemExpress) administered 2 h before LPS stimulation. For ROS scavenging, N‐acetyl‐L‐cysteine (NAC; 5 mM; HY‐B2015, MedChemExpress) was added 2 h before LPS stimulation.

### 2.10 Cell Viability Assay

The cytotoxic effect of Olaparib on THP‐1‐derived macrophages was assessed using a Cell Counting Kit‐8 (CCK‐8; HY‐K0301, MedChemExpress) according to the manufacturer's instructions. Differentiated macrophages were seeded in 96‐well plates at a density of 5 × 10^3^ cells per well and incubated overnight to allow cell adherence. Cells were then treated with increasing concentrations of Olaparib (0, 1, 5, 10, 20, and 50 μM) for 24 h. Following, 10 μL of CCK‐8 reagent was added to each well, and plates were incubated for 2 h at 37°C in a humidified 5% CO_2_ atmosphere. Absorbance was recorded at 450 nm using a SpectraMax i3x microplate reader. Cell viability was calculated as a percentage of the vehicle‐treated control group after background subtraction.

### 2.11 Lactate Dehydrogenase (LDH) Release Assay

Cytotoxicity was assessed by measuring LDH release into the culture supernatant using a commercial LDH Cytotoxicity Assay Kit (C0016, Beyotime) according to the manufacturer's instructions. THP‐1‐derived macrophages were seeded in 96‐well plates at a density of 5 × 10^4^ cells per well and incubated overnight for stabilization. After the indicated treatments, 100 μL of culture supernatant from each well was transferred to a fresh 96‐well plate. LDH working solution (100 μL) was then added to each well, followed by incubation for 30 min at 25°C in the dark. The reaction was terminated by adding 50 μL of stop solution, and absorbance was immediately measured at 490 nm using a SpectraMax i3x microplate reader. Cytotoxicity was calculated as follows: [(experimental release—spontaneous release) / (maximum release—spontaneous release)] × 100%.

### 2.12 NAD ^+^/NAPDH Detection Assay

The NAD^+^/NADH ratio was quantified using an NAD^+^/NADH colorimetric quantitation kit (K337‐100, BioVision) according to the manufacturer's instructions. For in vitro analysis, THP‐1‐derived macrophages (1 × 10^6^ cells) were harvested and lysed in 400 μL of NAD^+^/NADH extraction buffer supplemented with protease inhibitors. For tissue analysis, 30 mg of snap‐frozen lung tissue was homogenized in 600 μL of ice‐cold extraction buffer using a Tissuelyser LT system (85,600, Qiagen) at 30 Hz for 2 × 30 s. All samples were centrifuged at 12,000*g* for 5 min at 4°C, and the supernatants were collected for immediate analysis. For selective NAD^+^ measurement, 200 μL of each sample was incubated with NADH degradation buffer at 37°C for 15 min to remove NADH, followed by incubation with NAD^+^ detection mix for 30 min at 37°C. For NADH determination, parallel samples were processed without the degradation buffer. Absorbance was measured at 450 nm using a SpectraMax i3x microplate reader. NAD^+^ and NADH concentrations were calculated from standard curves and normalized to total protein content determined by BCA assay. The NAD^+^/NADH ratio was subsequently derived from the quantified nucleotide levels.

### 2.13 AMP/ATP Detection Assay

The AMP/ATP ratio was quantified using an ATP/ADP/AMP Assay Kit (MAK135, Sigma‐Aldrich) following an optimized protocol. Differentiated THP‐1 macrophages (5 × 10^5^ cells per well) were rapidly washed with ice‐cold PBS and lysed in 100 μL of 0.6 M perchloric acid containing 1 mM EDTA. After incubation on ice for 10 min, lysates were neutralized with 50 μL of 2 M KOH/0.3 M MOPS buffer (pH 7.4) and centrifuged at 12,000*g* for 10 min at 4°C. The resulting supernatants were collected for immediate analysis. ATP and AMP levels were measured according to the manufacturer's instructions. Briefly, 10 μL aliquots were used for ATP quantification based on luciferase‐mediated bioluminescence detection. For AMP determination, parallel 50 μL aliquots were treated with apyrase (5 μL) to degrade ATP, followed by enzymatic conversion for AMP measurement. Luminescence signals were recorded using a SpectraMax i3x microplate reader with a 5‐s integration time. Nucleotide concentrations were calculated from standard curves and normalized to total protein content determined by BCA assay. The AMP/ATP ratio was then calculated from the measured concentrations of both nucleotides.

### 2.14 Western Blot

Cultured cells and frozen tissue samples were lysed in radioimmunoprecipitation (RIPA) buffer (P0013B, Beyotime) supplemented with protease and phosphatase inhibitor cocktails. Protein concentrations were quantified using a BCA assay (P0012, Beyotime). Equal amounts of protein (30 μg per sample) were resolved on 10% sodium dodecyl sulfate–polyacrylamide gel electrophoresis (SDS‐PAGE) and subsequently transferred onto polyvinylidene difluoride (PVDF) membranes (IPVH00010, Millipore). Membranes were blocked with 5% non‐fat milk and incubated overnight at 4°C with primary antibodies targeting p‐AMPK (ab68206, 1:2000; Abcam), AMPK (ab32112, 1:1000; Abcam), p‐ULK1 (#89267, 1:1000; Cell Signaling Technology), ULK1 (#8054, 1:1000; Cell Signaling Technology), LC3 (ab192890, 1:2000; Abcam), p62 (ab314504, 1:1000; Abcam), NLRP3 (ab263899, 1:1000; Abcam), ASC (ab309497, 1:1000; Abcam), cleaved caspase‐1 (human, #4199; mouse, #89332; 1:1000; Cell Signaling Technology), IL‐1β (ab283818, 1:1000; Abcam), ZO‐1 (ab276131, 1:1000; Abcam), claudin‐5 (#49564, 1:1000; Cell Signaling Technology), VE‐cadherin (ab205336, 1:1000; Abcam), Bax (ab32503, 1:1000; Abcam), cleaved caspase‐3 (ab214430, 1:5000; Abcam), and GAPDH (ab8245, 1:1000; Abcam). Following primary antibody incubation, membranes were incubated with HRP‐conjugated secondary antibodies. Protein bands were visualized using an enhanced chemiluminescence (ECL) detection kit (WBKLS0500, Millipore) and imaged with a ChemiDoc MP System (Bio‐Rad). Densitometric analysis was performed using Image Lab software (v6.1).

### 2.15 Enzyme‐Linked Immunosorbent Assay (ELISA)

Concentrations of IL‐1β and IL‐18 in cell culture supernatants and BALF were quantified using commercial ELISA kits (Human IL‐1β ELISA Kit, PI305, Beyotime; Human IL‐18 ELISA Kit, PI558, Beyotime; Mouse IL‐1β ELISA Kit, PI301, Beyotime; Mouse IL‐18 ELISA Kit, PI553, Beyotime) according to optimized protocols. For in vitro analysis, supernatants from treated THP‐1‐derived macrophages were collected following centrifugation at 500*g* for 10 min at 4°C. For in vivo investigation, BALF samples were first centrifuged at 400*g* for 10 min to remove cells, followed by centrifugation at 10,000*g* to eliminate residual debris. Standards and samples (50 μL) were added to antibody‐precoated wells and incubated at 37°C for 2 h. This was followed by sequential incubation with biotinylated detection antibody and HRP, each for 1 h at 37°C. Color development was achieved using TMB substrate for 30 min at 37°C in the dark, and the reaction was terminated with 2 M H_2_SO_4_. Absorbance was measured at 450 nm using a SpectraMax i3x microplate reader. Cytokine concentrations were calculated from standard curves and normalized to total protein content determined by BCA assay.

### 2.16 Measurement of Autophagic Flux

Autophagic flux was quantitatively evaluated using an mRFP‐GFP‐LC3 tandem fluorescent reporter (HB‐AP2100001, Hanbio, Shanghai) in combination with confocal microscopy. THP‐1‐derived macrophages were seeded in 35‐mm glass‐bottom dishes at a density of 5 × 10^4^ cells per dish and transduced with mRFP‐GFP‐LC3 lentivirus (MOI = 20) in the presence of 8 μg/mL polybrene (C0351, Beyotime) for 24 h. Following transduction, cells were subjected to the indicated treatments and fixed with 4% paraformaldehyde. Fluorescent images were acquired using a Zeiss LSM 900 confocal microscope (Carl Zeiss). Autophagic flux was assessed by quantifying the number of GFP^+^mRFP^+^ puncta (autophagosomes) and GFP^−^mRFP^+^ puncta (autolysosomes) per cell using ImageJ software (v1.53). Fifteen randomly selected cells per group were analyzed, and results were expressed as the ratio of mRFP^+^GFP^−^ puncta to total LC3 puncta.

### 2.17 Measurement of Malondialdehyde (MDA)

MDA levels were quantified using a thiobarbituric acid (TBA)‐based Lipid Peroxidation MDA Assay Kit (S0131M, Beyotime) in accordance with the manufacturer's instructions. For in vitro analysis, THP‐1‐derived macrophages (5 × 10^6^ cells) were washed with ice‐cold PBS and lysed in 500 μL RIPA buffer supplemented with 0.1% butylated hydroxytoluene (BHT) to minimize artificial oxidation. For tissue analysis, 30 mg of snap‐frozen lung samples were homogenized in 600 μL PBS containing 0.1% BHT using a Tissuelyser LT system. Cell lysates and tissue homogenates were centrifuged at 12,000*g* for 15 min at 4°C, and the supernatants were collected. Protein concentrations were determined using a BCA assay, and samples were normalized to 1 mg/mL. Following, 100 μL of each sample was combined with 200 μL of TBA working solution and incubated at 95°C for 60 min. After cooling to room temperature, absorbance was measured at 532 nm using a SpectraMax i3x microplate reader. MDA concentrations were derived from a standard calibration curve and normalized to total protein content.

### 2.18 Intracellular ROS Detection

Intracellular ROS levels were quantified using the fluorogenic probe 2′,7′‐dichlorodihydrofluorescein diacetate (DCFH‐DA; S0033S, Beyotime). THP‐1‐derived macrophages were seeded in black‐walled 96‐well imaging plates at a density of 5 × 10^3^ cells per well. Following treatment, cells were incubated with 10 μM DCFH‐DA prepared in serum‐free RPMI‐1640 medium at 37°C for 30 min in the dark. After incubation, cells were washed twice with pre‐warmed PBS and maintained in phenol red‐free medium. Fluorescence images were acquired using an ImageXpress Pico Automated Cell Imaging System (Molecular Devices), with a minimum of 10 fields captured per well. DCF fluorescence intensity was quantified using CellReporterXpress software (v2.5). Data were expressed as mean fluorescence intensity (MFI) per cell following background subtraction.

### 2.19 Mitochondrial ROS Detection

Mitochondrial superoxide levels were quantified using the MitoSOX Red mitochondrial superoxide indicator (M36008, Thermo Fisher Scientific). THP‐1‐derived macrophages were seeded in black‐walled 96‐well imaging plates at a density of 5 × 10^3^ cells per well. Following treatment, cells were incubated with 5 μM MitoSOX Red reagent prepared in serum‐free RPMI‐1640 medium at 37°C for 30 min in the dark. After incubation, cells were washed twice with pre‐warmed PBS and maintained in phenol red‐free medium. Fluorescence images were acquired immediately using an ImageXpress Pico Automated Cell Imaging System. Quantitative analysis was performed using CellReporterXpress software (v2.5) to determine the MFI of MitoSOX Red per cell. Data were normalized to the control group.

### 2.20 Statistical Analysis

Statistical analyses were conducted using GraphPad Prism software (version 9.0.0; GraphPad Software Inc.). Quantitative data derived from at least three independent experiments were presented as mean ± standard deviation. Comparisons among multiple groups were performed using one‐way analysis of variance followed by Tukey's post hoc test to evaluate statistical significance. A *p*‐value < 0.05 was considered statistically significant.

## 3 Results

### 3.1 Olaparib Induces Metabolic Reprogramming in Macrophages and Activates AMPK‐Mediated Autophagic Flux

CCK‐8 assay demonstrated that Olaparib (0–50 μM) did not induce significant cytotoxicity in THP‐1‐derived macrophages, indicating that observed effects were independent of cell viability (Figure 1A). Assessment of the NAD^+^/NADH ratio showed no significant alteration upon LPS/ATP stimulation alone; however, co‐treatment with Olaparib (5, 10, and 20 μM) resulted in a dose‐dependent reduction under LPS/ATP conditions (Figure 1B). Analysis of the AMP/ATP ratio indicated an increasing trend following LPS/ATP stimulation relative to the control group, although the change did not reach statistical significance. In comparison, Olaparib treatment significantly elevated the AMP/ATP ratio in a dose‐dependent manner (Figure 1C). These metabolic alterations induced by Olaparib were not reversed by the AMPK inhibitor compound C (10 μM) (Figure 1B–C). To further examine downstream signaling events, immunoblot analysis was performed. Olaparib treatment increased the p‐AMPK/AMPK and p‐ULK1/ULK1 ratios, enhanced the LC3‐II/LC3‐I ratio, and reduced p62 protein levels in LPS/ATP‐treated macrophages. These effects were attenuated upon co‐treatment with compound C (Figure 1D–G). Evaluation of autophagic flux using the mRFP‐GFP‐LC3 tandem reporter showed that LPS/ATP stimulation led to the accumulation of yellow puncta (autophagosomes) without a corresponding increase in red puncta (autolysosomes), indicating impaired autophagic flux. Olaparib treatment significantly increased red puncta formation, consistent with restoration of autophagic flux. This effect was suppressed by compound C (Figure 1H–I).

**FIGURE 1:**
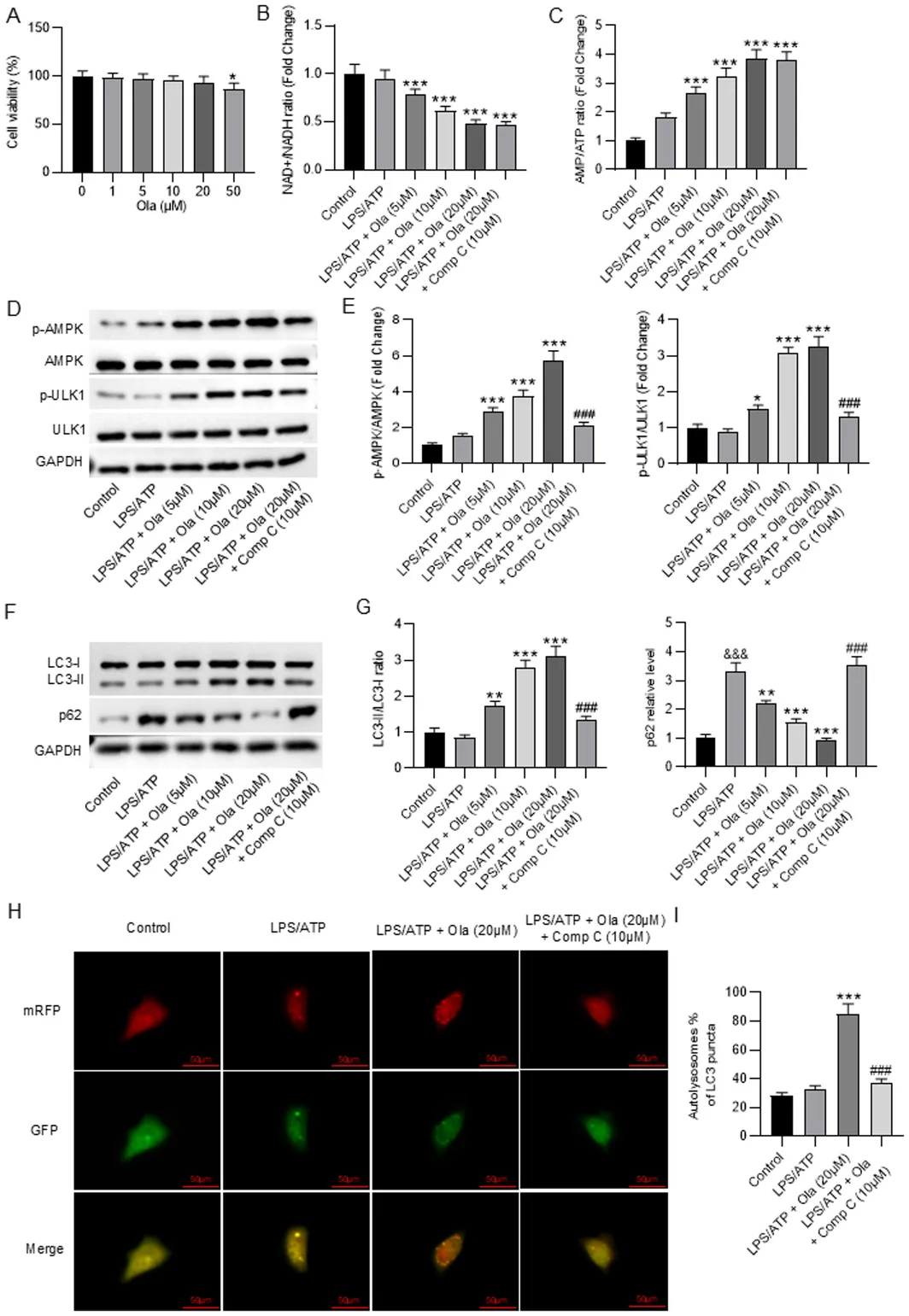
Olaparib activates AMPK‐dependent metabolic and autophagic responses in macrophages. (A) Cell viability of PMA‐differentiated THP‐1 macrophages treated with Olaparib (0–50 μM) was assessed using the CCK‐8 assay. *p < 0.05 versus control group. (B) NAD^+^/NADH ratio was measured in PMA‐differentiated THP‐1 macrophages under control, LPS/ATP, LPS/ATP + Olaparib (5 μM), LPS/ATP + Olaparib (10 μM), LPS/ATP + Olaparib (20 μM), and LPS/ATP + Olaparib (20 μM) + compound C (10 μM) conditions. (C) AMP/ATP ratio in THP‐1 macrophages across the indicated experimental groups. (D–E) Protein expression levels of p‐AMPK, AMPK, p‐ULK1, and ULK1 in THP‐1 macrophages assessed by Western blot. (F–G) LC3‐II/LC3‐I ratio and p62 protein levels determined by Western blot analysis. (H–I) Autophagic flux in control, LPS/ATP, LPS/ATP + Olaparib (20 μM), and LPS/ATP + Olaparib (20 μM) + compound C (10 μM) groups evaluated using the mRFP–GFP–LC3 tandem fluorescent reporter system. Data are presented as mean ± standard deviation from three independent experiments. ^&&&^ *p* < 0.001 versus control group; *p < 0.05, **p < 0.01, ***p < 0.001 versus LPS/ATP group; ^###^ *p* < 0.001 versus LPS/ATP + Olaparib (20 μM) group.

### 3.2 Olaparib Attenuates Oxidative Stress Damage and NLRP3 Inflammasome Activation via Autophagy Induction in LPS/ATP‐Induced Macrophages

The effect of Olaparib on oxidative stress and NLRP3 inflammasome activation was assessed in LPS/ATP‐stimulated macrophages. LPS/ATP exposure significantly elevated both intracellular and mitochondrial ROS levels, whereas treatment with Olaparib or NAC (a ROS scavenger) significantly reduced this increase. Co‐treatment with 3‐MA, an autophagy inhibitor, reversed the suppressive effect of Olaparib on ROS accumulation, indicating an autophagy‐dependent mechanism (Figure 2A–D). MDA levels, indicative of lipid peroxidation, were measured using a commercial assay kit. LPS/ATP stimulation induced a significant increase in MDA production, which was reduced by Olaparib and NAC treatment. This reduction was negated upon 3‐MA co‐treatment, further supporting the involvement of autophagy in mediating the antioxidant effect of Olaparib (Figure 2E). LDH release, reflecting NLRP3 inflammasome‐mediated pyroptotic cell death, was significantly increased following LPS/ATP stimulation. This effect was attenuated by Olaparib or NAC, while 3‐MA co‐treatment restored LDH release levels, indicating reversal of Olaparib‐mediated protection (Figure 2F). ELISA analysis demonstrated that LPS/ATP stimulation significantly increased the secretion of IL‐1β and IL‐18. Treatment with Olaparib or NAC reduced the levels of these pro‐inflammatory cytokines, whereas co‐treatment with 3‐MA diminished the inhibitory effect of Olaparib (Figure 2G). Western blot analysis showed that LPS/ATP stimulation increased the protein expression of NLRP3, ASC, cleaved caspase‐1, and IL‐1β. These elevations were reduced following treatment with Olaparib or NAC. The presence of 3‐MA reversed the suppressive effect of Olaparib on these inflammasome‐related proteins, indicating that autophagy plays a critical role in regulating NLRP3 inflammasome activation under these conditions (Figure 2H–I).

**FIGURE 2:**
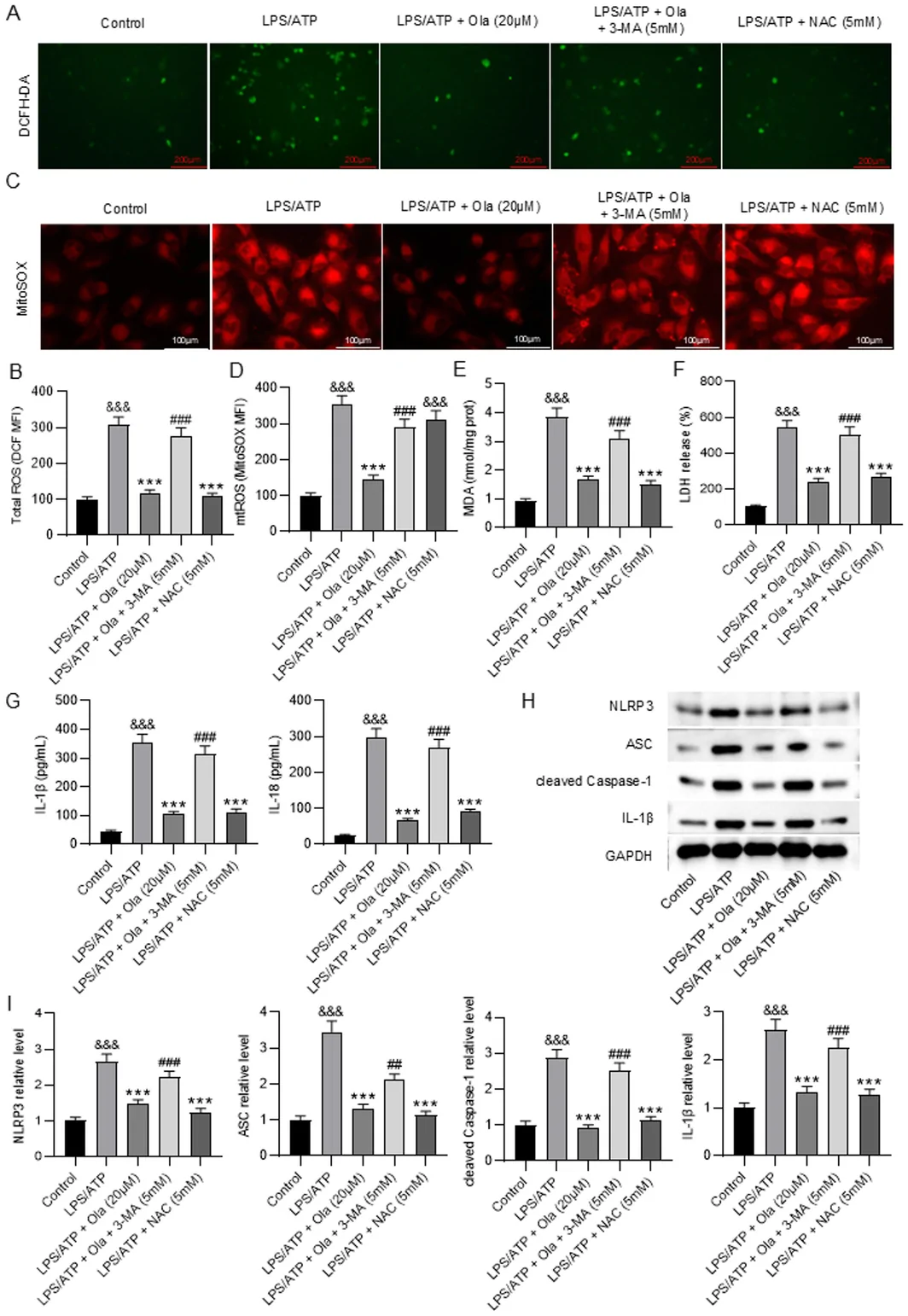
Olaparib suppresses oxidative stress and NLRP3 activation via autophagy in macrophages. (A–B) Intracellular ROS levels were assessed by DCFH‐DA staining in control, LPS/ATP, LPS/ATP + Olaparib (20 μM), LPS/ATP + Olaparib (20 μM) + 3‐MA (5 mM), and LPS/ATP + Olaparib (20 μM) + NAC (5 mM) groups. (C–D) Mitochondrial ROS levels were evaluated using MitoSOX staining. (E) Measurement of MDA levels. (F) LDH activity assay. (G) ELISA‐based quantification of IL‐1β and IL‐18 levels. (H–I) Western blot analysis of NLRP3, ASC, cleaved caspase‐1, and IL‐1β protein expression. Data are presented as mean ± standard deviation from three independent experiments. ^&&&^ *p* < 0.001 versus control group; ***p < 0.001 versus LPS/ATP group; ^##^ *p* < 0.01, ^###^ *p* < 0.001 versus LPS/ATP + Olaparib (20 μM) group.

### 3.3 Olaparib Ameliorates Oxidative Stress and NLRP3 Inflammasome Activation in ALI Mice via AMPK‐Mediated Autophagic Flux

The in vivo therapeutic efficacy of Olaparib was investigated using the LPS‐induced ALI model in 3–4‐week‐old mice. Analysis of lung tissues displayed that treatment with Olaparib (100 mg/kg) significantly reduced the NAD^+^/NADH ratio in LPS‐exposed animals (Figure 3A). Western blot analysis of lung homogenates revealed that Olaparib elevated the p‐AMPK/AMPK ratio while decreasing p62 protein levels in a dose‐dependent manner. These molecular effects were attenuated by co‐administration of compound C, as indicated by a reduction in the p‐AMPK/AMPK and LC3‐II/LC3‐I ratios, along with an increase in p62 expression (Figure 3B–C). Immunohistochemical assessment showed that LPS exposure significantly enhanced 4‐HNE expression in lung tissues, which was significantly mitigated following Olaparib treatment; however, compound C co‐treatment restored 4‐HNE levels toward those observed in the LPS group (Figure 3D–E). In agreement with these findings, quantification of MDA levels confirmed that Olaparib significantly suppressed LPS‐induced lipid peroxidation, whereas this inhibitory effect was abolished upon compound C administration (Figure 3F). Furthermore, ELISA‐based measurement of BALF indicated that LPS challenge significantly increased IL‐1β and IL‐18 concentrations, which were significantly reduced by Olaparib treatment and reinstated following compound C co‐treatment (Figure 3G). Western blot analysis further demonstrated that Olaparib significantly suppressed LPS‐induced upregulation of NLRP3, ASC, and cleaved caspase‐1 protein levels, whereas compound C reversed these inhibitory effects and restored inflammasome activation markers (Figure 3H–I).

**FIGURE 3:**
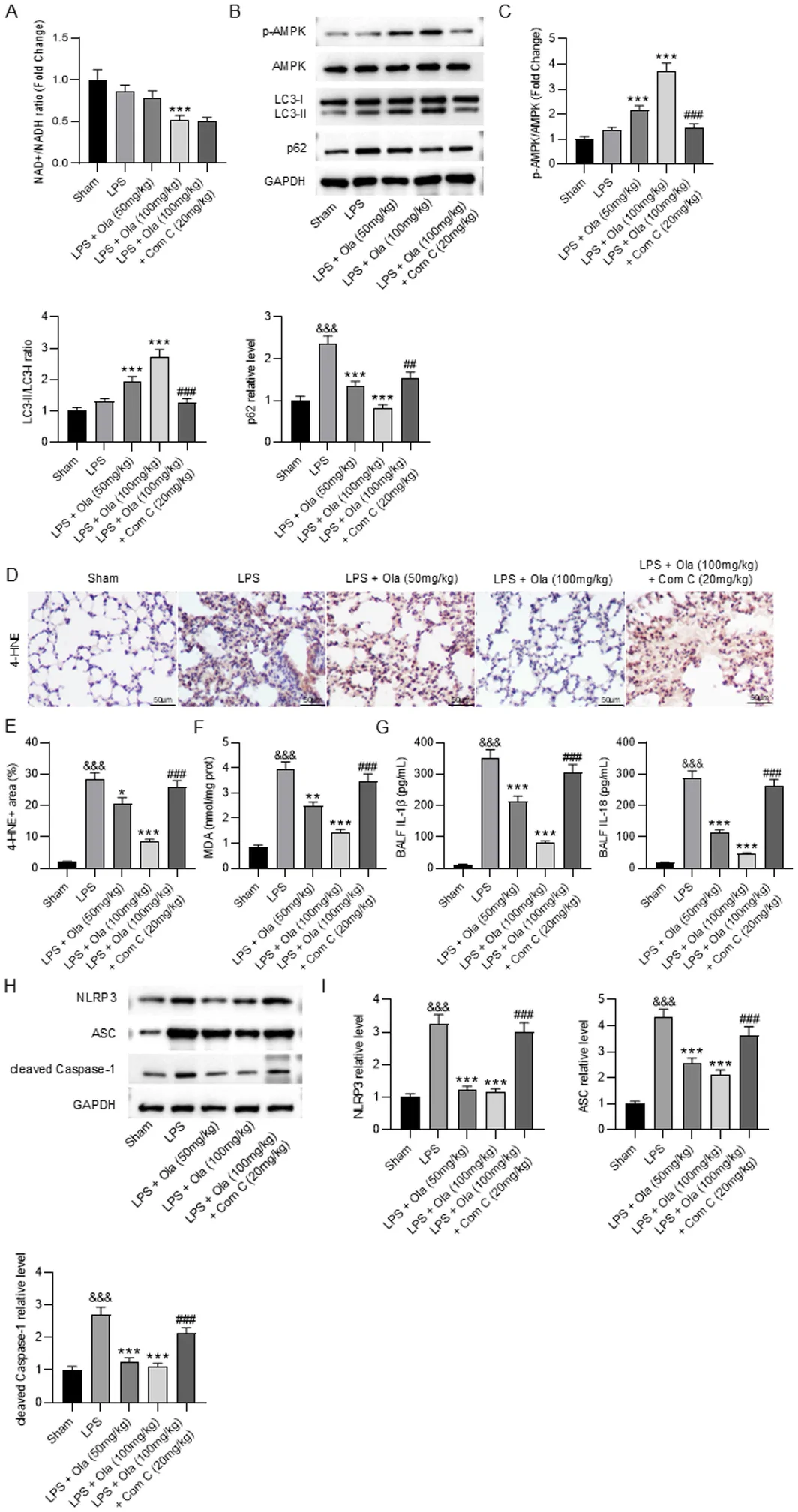
Olaparib suppresses oxidative stress and NLRP3 activation via AMPK‐dependent autophagy in ALI mice. (A) NAD^+^/NADH ratio in lung tissues from control, LPS, LPS + Olaparib (50 mg/kg), LPS + Olaparib (100 mg/kg), and LPS + Olaparib (100 mg/kg) + compound C (20 mg/kg) groups. (B–C) Western blot analysis of p‐AMPK/AMPK and LC3‐II/LC3‐I ratios, along with p62 protein levels in lung tissues. (D–E) Immunohistochemical staining and quantification of 4‐HNE expression. (F) Measurement of MDA levels in lung tissues. (G) Levels of IL‐1β and IL‐18 in BALF determined by ELISA. (H–I) Western blot analysis of NLRP3, ASC, and cleaved caspase‐1 protein expression. Data are presented as mean ± standard deviation (*n* = 6). ^&&&^ *p* < 0.001 versus sham group; *p < 0.05, **p < 0.01, ***p < 0.001 versus LPS group; ^##^ *p* < 0.01, ^###^ *p* < 0.001 versus LPS + Olaparib (100 mg/kg) group.

### 3.4 Olaparib Attenuates Pathological Injury and Inflammatory Cell Infiltration in the Lungs of ALI Mice

Histological examination using H&E staining demonstrated significant structural damage in the lung tissues of the LPS group, characterized by alveolar congestion, hemorrhage, interstitial edema, and extensive inflammatory cell infiltration. Treatment with Olaparib (50 and 100 mg/kg) significantly alleviated these LPS‐induced pathological alterations in a dose‐dependent manner. This protective effect was abrogated upon co‐administration of compound C (Figure 4A–B). LPS exposure significantly increased the lung wet/dry weight ratio, reflecting the development of pulmonary edema, whereas Olaparib treatment dose‐dependently reduced this elevation. Compound C co‐treatment reversed Olaparib's ability to reduce the wet/dry ratio (Figure 4C). Immunohistochemical analysis further revealed a significant increase in F4/80‐positive macrophage infiltration in LPS‐treated lungs compared with controls, which was significantly diminished following Olaparib administration in a dose‐dependent manner. This reduction was negated by compound C co‐treatment (Figure 4D–E). Colorimetric assays demonstrated that Olaparib significantly suppressed LPS‐induced MPO activity in both BALF and lung tissues, whereas compound C abolished this inhibitory effect (Figure 4F–G).

**FIGURE 4:**
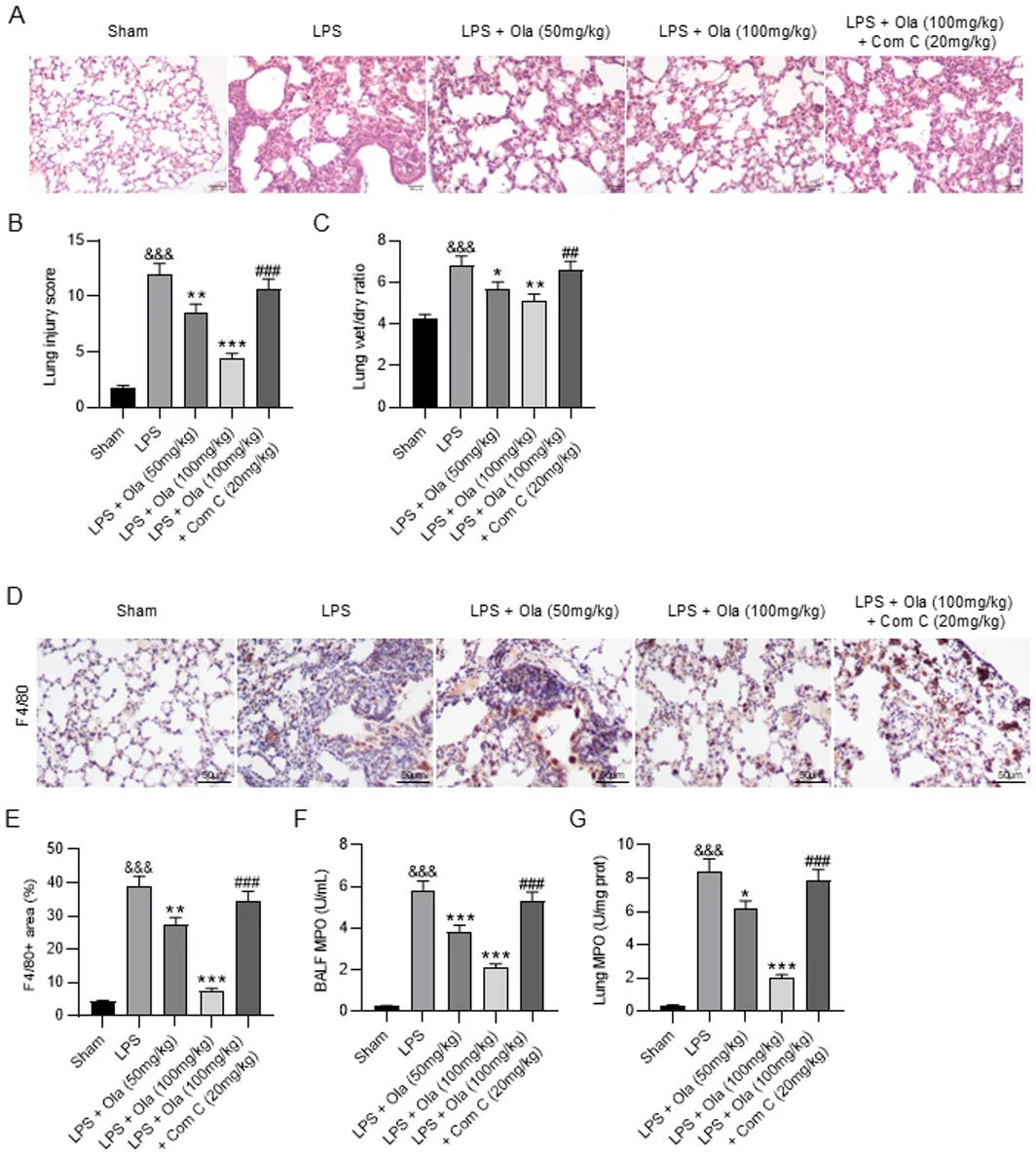
Olaparib alleviates pulmonary injury and inflammatory cell infiltration in ALI mice. (A) Representative H&E‐stained lung sections from control, LPS, LPS + Olaparib (50 mg/kg), LPS + Olaparib (100 mg/kg), and LPS + Olaparib (100 mg/kg) + compound C (20 mg/kg) groups. (B) Quantification of lung injury score. (C) Lung wet/dry (W/D) ratio assessment. (D–E) Immunohistochemical staining and quantification of F4/80‐positive areas in lung tissues. (F) MPO activity in BALF. (G) MPO activity in lung tissue homogenates. Data are presented as mean ± standard deviation (*n* = 6). ^&&&^ *p* < 0.001 versus sham group; *p < 0.05, **p < 0.01, ***p < 0.001 versus LPS group; ^##^ *p* < 0.01, ^###^ *p* < 0.001 versus LPS + Olaparib (100 mg/kg) group.

### 3.5 Olaparib Alleviates Lung Barrier Dysfunction and Apoptosis in LPS‐Induced ALI Mice

Finally, the effect of Olaparib on LPS‐induced pulmonary barrier dysfunction and apoptosis was evaluated. Western blot analysis showed that LPS significantly decreased the expression of key endothelial junctional proteins, including ZO‐1, claudin‐5, and VE‐cadherin, in lung tissues. Olaparib treatment significantly restored the expression of these proteins, whereas co‐administration of compound C reversed this restorative effect (Figure 5A–B). TUNEL staining revealed a significant increase in TUNEL‐positive area in the LPS group relative to controls, which was significantly reduced following Olaparib treatment. This anti‐apoptotic effect was diminished upon compound C co‐treatment (Figure 5C–D). Western blot confirmed that Olaparib suppressed LPS‐induced upregulation of pro‐apoptotic markers, including Bax and cleaved caspase‐3, whereas compound C reversed the inhibitory effects of Olaparib on these apoptotic proteins (Figure 5E–F).

**FIGURE 5:**
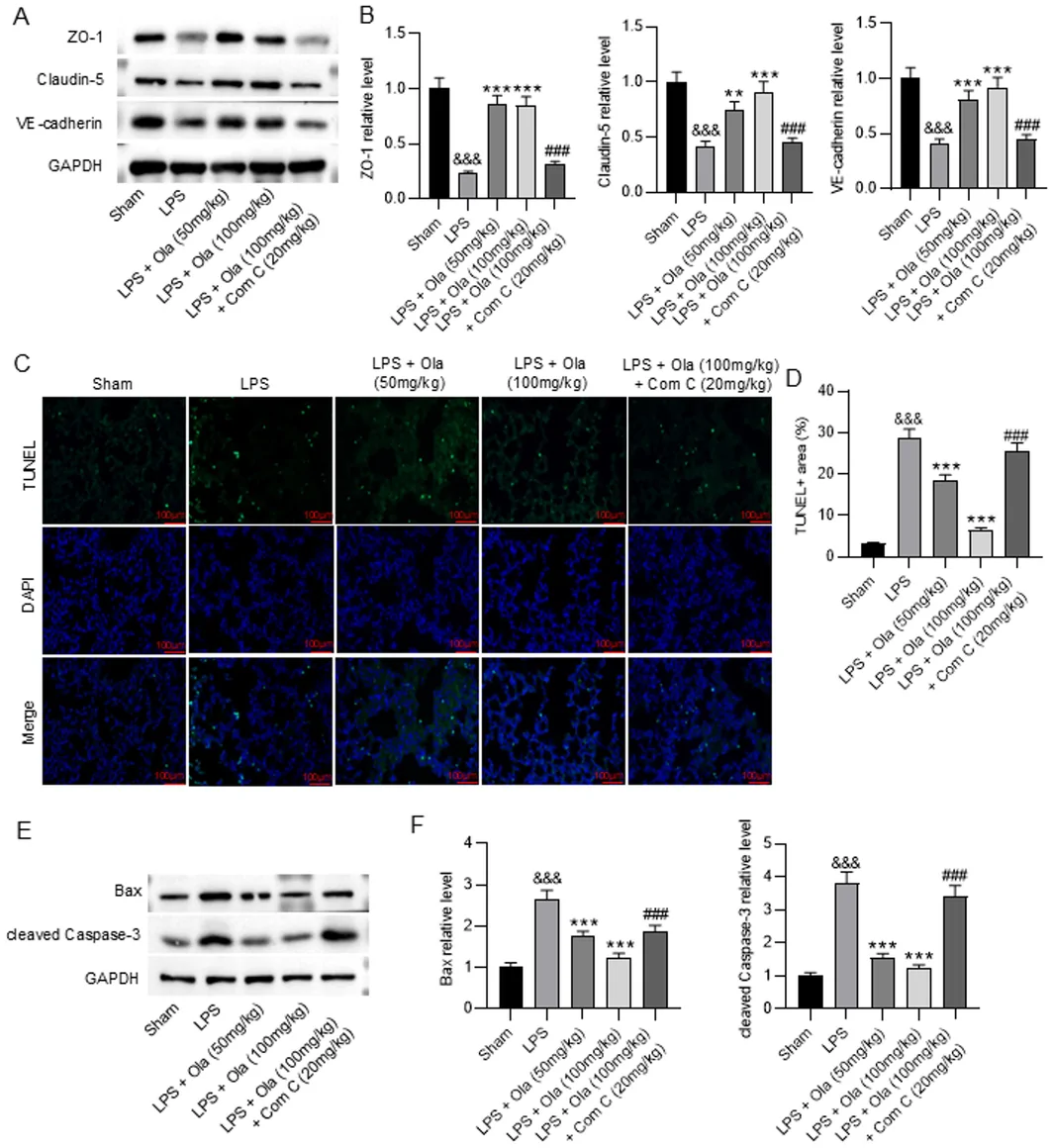
Olaparib reduces lung barrier dysfunction and apoptosis in ALI mice. (A–B) Protein expression levels of ZO‐1, claudin‐5, and VE‐cadherin in lung tissues from control, LPS, LPS + Olaparib (50 mg/kg), LPS + Olaparib (100 mg/kg), and LPS + Olaparib (100 mg/kg) + compound C (20 mg/kg) groups were analyzed by Western blot. (C–D) TUNEL staining and quantification of TUNEL‐positive area in lung tissues. (E–F) Western blot analysis of Bax and cleaved caspase‐3 protein expression in lung tissues. Data are presented as mean ± standard deviation (*n* = 6). ^&&&^ *p* < 0.001 versus sham group; **p < 0.01, ***p < 0.001 versus LPS group; ^###^ *p* < 0.001 versus LPS + Olaparib (100 mg/kg) group.

## 4 Discussion

Our findings indicate that Olaparib provides dose‐dependent protection against LPS‐induced pulmonary injury in 3–4‐week‐old C57BL/6J mice, as reflected by reduced alveolar hemorrhage, decreased inflammatory cell infiltration, and improved integrity of the pulmonary barrier. The protective effects of Olaparib are mediated through metabolic reprogramming with activation of the AMPK/ULK1 signaling pathway, leading to enhanced autophagic flux, alongside suppression of oxidative stress and inhibition of NLRP3 inflammasome activation.

Our study demonstrates that Olaparib induces a metabolic shift characterized by a reduced NAD^+^/NADH ratio and an elevated AMP/ATP ratio in LPS/ATP‐stimulated macrophages, resulting in promotion of AMPK activation. Although this finding appears to differ from the canonical role of PARP inhibition in preserving cellular energy by limiting PARP‐1–mediated NAD^+^ and ATP depletion during severe genotoxic stress [24], this divergence likely reflects a context‐dependent metabolic response. The NAD^+^/NADH ratio serves as a key indicator of cellular redox balance and metabolic flux, in which NAD^+^ functions as an essential electron acceptor in redox reactions, whereas NADH represents its reduced form [25]. The AMP/ATP ratio is a central determinant of cellular energy status, where increased AMP levels reflect energetic stress and act as a primary trigger for AMPK activation [26]. In classical models of extensive DNA damage, PARP‐1 hyperactivation drives profound consumption of NAD^+^ and ATP, and PARP inhibitors such as Olaparib prevent this energetic collapse [27]. However, in the present inflammatory stress model (LPS/ATP), lacking extensive DNA damage, where PARP‐1 activation is likely more moderate, Olaparib appears to elicit a regulated metabolic reprogramming. Under these conditions, Olaparib‐mediated modulation of NAD^+^ metabolism and the resulting shift in the AMP/ATP ratio are not indicative of pathological energy failure but rather reflect a controlled metabolic alteration that enhances activation of the AMPK energy‐sensing pathway [28]. A key observation supporting this hierarchical model is that pharmacological inhibition of AMPK with compound C attenuated the protective effects of Olaparib on autophagy, oxidative stress, and NLRP3 inflammasome activation, yet failed to restore the Olaparib‐induced reduction in the NAD^+^/NADH ratio (Figure 1B). These findings suggest that the modulation of NAD^+^ metabolism occurs upstream as a direct consequence of PARP inhibition, whereas activation of the AMPK–autophagy pathway represents a downstream effector pathway mediating cytoprotection. Therefore, the shift in the NAD^+^/NADH balance is not sufficient to drive protection independently; rather, it establishes a metabolic environment that enables subsequent AMPK activation. This metabolic reprogramming initiates a signaling cascade characterized by enhanced autophagic flux and suppression of NLRP3 inflammasome activity, therefore contributing to attenuation of inflammatory and oxidative injury in pediatric ALI.

AMPK is a central metabolic regulator required for cellular energy homeostasis during stress and is deeply involved in inflammatory control and metabolic reprogramming [29, 30]. Beyond its canonical role in energy sensing, AMPK regulates autophagy by directly phosphorylating ULK1 at key sites, hence initiating autophagosome biogenesis [31]. Consistent with this mechanism, the present data demonstrate that Olaparib‐mediated AMPK activation enhances ULK1 phosphorylation, increases LC3‐II conversion, and promotes p62 degradation. Olaparib restored autophagic flux impaired by LPS/ATP, as reflected by restored autophagosome maturation and improved autophagosome–lysosome fusion, indicating recovery of lysosomal degradative capacity. This restoration of functional autophagy represents a key determinant of its cytoprotective effects against oxidative injury. While the broad‐spectrum antioxidant NAC reduced total intracellular ROS and lipid peroxidation, as indicated by decreased MDA levels, it had limited efficacy in suppressing mitochondrial ROS under the experimental conditions. This differential response underscores the spatial compartmentalization of oxidative stress regulation, in which NAC primarily acts within the cytosolic environment due to restricted mitochondrial bioavailability, whereas efficient mitochondrial ROS clearance requires selective elimination of damaged mitochondria via mitophagy [32, 33]. The Olaparib‐induced enhancement of autophagic flux therefore represents a more integrated antioxidant mechanism. In this process, damaged mitochondria, the principal source of mitochondrial ROS, are selectively removed, while oxidized proteins and lipid peroxidation products are concurrently degraded.

Excessive ROS and oxidized lipids are recognized activators of the NLRP3 inflammasome, although they operate through partially distinct mechanisms [34]. The present findings indicate that different antioxidant interventions can converge on suppression of NLRP3 activation via mechanistically divergent routes. NAC attenuates inflammasome activation predominantly by scavenging cytosolic ROS and limiting lipid peroxidation, reducing upstream inflammatory signals required for NLRP3 priming and activation. Olaparib exerts its effects through a more upstream regulatory mechanism by promoting mitophagy, hence removing damaged mitochondria, the principal source of mitochondrial ROS, while also enhancing autophagic degradation of oxidatively modified macromolecules. This mechanistic distinction may explain why both interventions effectively suppress NLRP3 inflammasome assembly and downstream maturation of IL‐1β and IL‐18 despite differing impacts on mitochondrial ROS regulation [35, 36]. The autophagy dependence of Olaparib's effects was further substantiated by 3‐MA treatment, which restored NLRP3 inflammasome activation. Consistent with these findings, previous reports have demonstrated that Olaparib suppresses NLRP3 inflammasome activity while maintaining mitochondrial integrity [37]. These results highlight a multifaceted mechanism of action, wherein Olaparib modulates interconnected inflammatory and metabolic pathways. Together with its well‐established safety profile [38], these observations support its potential as a therapeutic candidate for ALI. It may act through coordinated regulation of the oxidative stress–inflammation pathway via activation of the AMPK–autophagy signaling pathway.

The therapeutic relevance of Olaparib in pediatric ALI is further supported by the developmental susceptibility of the immature lung. Compared with adults, the pediatric lung shows reduced baseline activity of key antioxidant enzymes, including catalase (CAT) and superoxide dismutase (SOD), along with potentially less efficient mitophagic capacity [39]. This developmental immaturity increases vulnerability to ROS‐mediated injury during ALI/ARDS. In this context, Olaparib‐mediated activation of AMPK‐dependent mitophagy and autophagy may compensate for these underdeveloped endogenous defense systems, as a result, enhancing cellular resilience in the pediatric lung. Furthermore, alveolar–capillary barrier integrity, which relies on coordinated assembly of tight and adherens junctions, continues to mature postnatally [40]. Proper expression of junctional proteins such as ZO‐1, claudin‐5, and VE‐cadherin is essential for maintaining barrier function, yet these components may be developmentally immature or disrupted during ALI. The Olaparib treatment significantly increased the expression of ZO‐1, claudin‐5, and VE‐cadherin in injured lung tissue, an effect diminished by AMPK inhibition with compound C. These findings suggest that Olaparib not only preserves endothelial barrier integrity but may also facilitate structural repair processes through AMPK‐dependent signaling, offering a potential advantage in promoting recovery in the developing pediatric lung.

Some limitations of this study should be acknowledged. A major limitation is the use of compound C as a pharmacological AMPK inhibitor, which is associated with known off‐target effects on multiple kinases. Therefore, future studies using more selective pharmacological inhibitors or genetic strategies are required to more definitively establish the role of AMPK signaling. These may include AMPK α1/α2 knockdown or conditional knockout models. A further consideration relates to translational applicability. Although Olaparib‐induced metabolic modulation appears to confer protection by triggering AMPK‐dependent adaptive responses in the acute injury setting, sustained or excessive ATP depletion in metabolically active pediatric tissues may pose a risk to essential cellular functions. Therefore, the long‐term safety profile and therapeutic window of this approach in the developing organism require systematic evaluation. Finally, while the juvenile mouse model captures key pathological features of pediatric ALI, species‐specific differences in immune responses and drug metabolism may limit direct extrapolation to humans. Validation in more physiologically relevant systems, including human primary lung cells, ex vivo tissues, or organoid models, will be necessary before clinical translation.

In conclusion, this study identifies a novel mechanism through which Olaparib attenuates pediatric ALI by inducing metabolic reprogramming that activates AMPK‐dependent autophagic flux. This pathway suppresses oxidative stress and NLRP3 inflammasome activation, ultimately restoring alveolar–capillary barrier integrity and reducing inflammation and apoptosis (Figure 6). These results establish a mechanistic framework supporting further evaluation of Olaparib as a potential therapeutic strategy for pediatric ALI.

**FIGURE 6:**
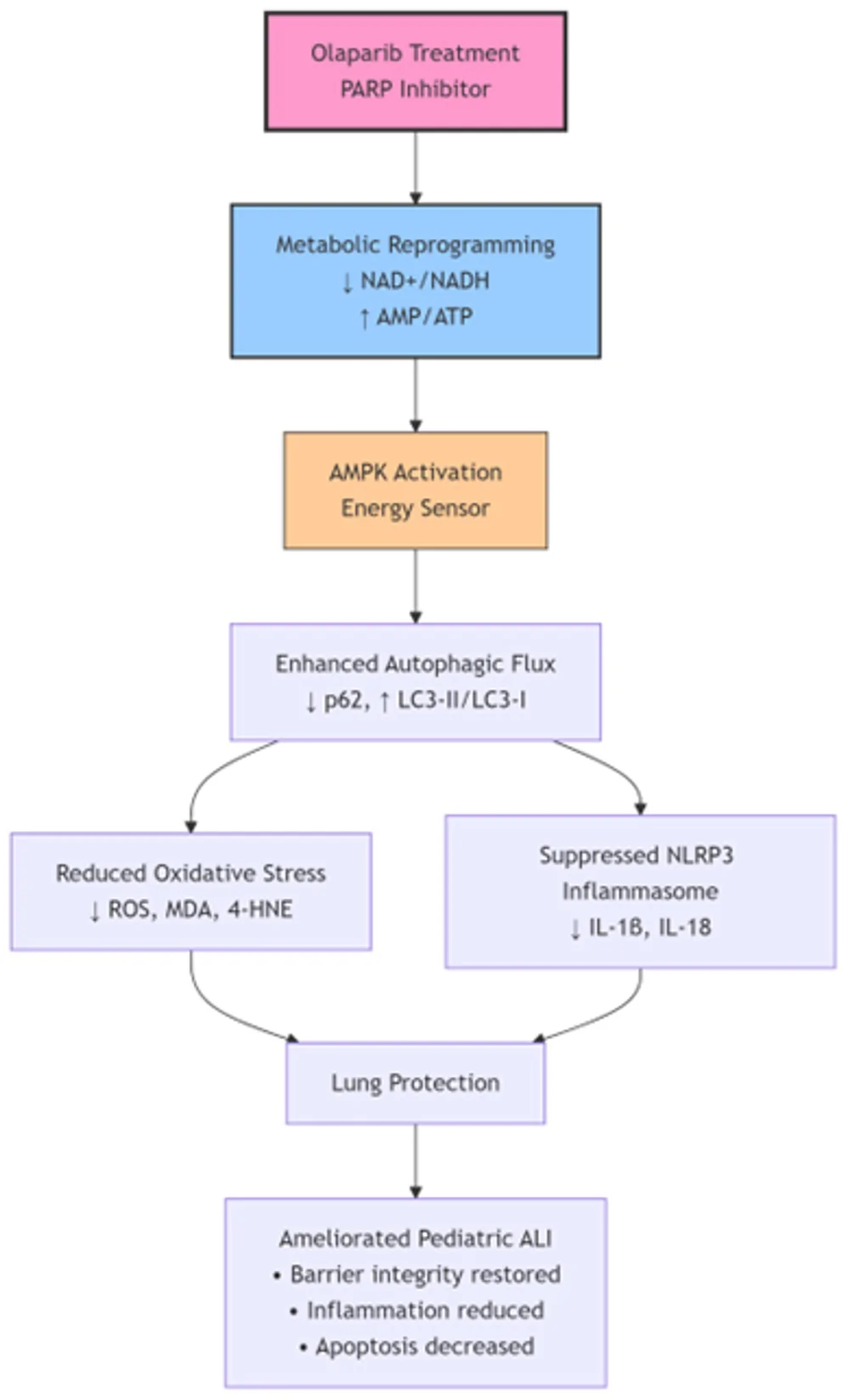
Proposed mechanism of Olaparib‐mediated protection in pediatric ALI. Olaparib ameliorates pediatric ALI by inducing metabolic reprogramming that activates AMPK‐dependent autophagic flux. This leads to suppression of oxidative stress and NLRP3 inflammasome activation, restoring alveolar–capillary barrier integrity and reducing inflammation and apoptosis.

## Ethics Statement

All animal procedures were reviewed and approved by the Ethical Committee of Children's Hospital of Soochow University (No. 2025CS110) and conducted in accordance with NIH Guide for the Care and Use of Laboratory Animals.

## Conflicts of Interest

The authors declare no conflicts of interest.

## Data Availability

The data that support the findings of this study are available from the corresponding author upon reasonable request.
